# The complete chloroplast genome of new alien species, *Celosia trigyna* L. (Amaranthaceae), in China

**DOI:** 10.1080/23802359.2019.1643802

**Published:** 2019-07-22

**Authors:** Han Xu, Xiaoyan Jing

**Affiliations:** Institute of plant quarantine, Chinese Academy of Inspection and Quarantine, Beijing, China

**Keywords:** Chloroplast genome, *Celosia trigyna*, China, Amaranthaceae, Caryophyllales

## Abstract

The first complete chloroplast genome sequences of new alien weeds in Guangdong, Celosia, were reported in this study. The *C. trigyna* plastome was 152,089 bp long, with the large single copy (LSC) region of 83,716 bp, the small single copy (SSC) region of 17,251 bp, and two inverted repeat (IR) regions of 25,561 bp. The plastome contained 130 genes, including 85 proteincoding, eight ribosomal RNA, and 37 transfer RNA genes. The overall GC content was 36.9%. Phylogenetic analysis of 21 representative plastomes within the order Caryophyllales suggests that C. trigyna is closely related to the species in genus *Amaranthus*.

The genus *Celosia* L. belongs to family Amaranthaceae and consists of about 45 species of perennial shrubs, subshrubs, or annual herbs. The genus is native to tropical America and Africa mostly (Flora of North America Editorial Committee 2015). *Celosia argentea* L. and *Celosia trigyna* are the most widely distributed species (GBIF (Global Invasive Species Database) Secretariat, 2017), especially *C. argentea* has dispersed all over the world as an ornamental and vegetable plant (NRC 2006). *Celosia trigyna* occurs almost throughout tropical Africa as a weed (Holm et al. 1997). Sometimes, it is served as a leafy vegetable and traditional medicine in parts of Africa (Gbile 1983). In addition, it has been introduced to the USA as an occasional weed (Flora of North America Editorial Committee 2015). Now, several individuals have been monitoring an oil processing plant near the southern ports in China. The impacts on the environments and crops of this alien weed should be noticed and estimated in the future. On the aspects of phylogenetic relationship, the genus *Celosia* has closer with *Hermbstaedtia*, *Pleuropetalum*, *Deeringia* in the Amaranthacea subfamily Amaranthoideae based on the *mat*K and *rbc*L analysis (Kadereit et al. 2003; Ogundipe and Chase 2009). Under the genus, the similar species of *C. trigyna* are *Celosia globosa* Schinz, *Celosia isertii* C.C.Towns., *Celosia leptostachya* Benth. and so on, and in itself has atleast three varieties (Townsend 1975). Thus, out of a new perspective aspect, discovering the taxonomic status of *C. trigyna* and phylogenetic relationships within the genus, and its phylogenetic position within Amaranthaceae is necessary and essential.

Total DNA (Voucher specimen: 23.018736°N, 113.535734°E, WuHR12) was isolated using the Plant Genomic DNA Kit (Tiangen Biotech Co., China) and sequenced by the Illumina HiSeq2500 platform (Novogene, Beijing, China). A total of 1,165,803 paired-end reads were assembled to *Amaranthus hypochondriacus* (GenBank accession nr: MG836505) to produce contigs using Geneious assembler (Biomatters, Auckland, New Zealand).

The total plastome length of *C. trigyna* (MN057637) was 152,089 bp, with a large single copy (LSC; 83,716 bp), small single copy (SSC; 17,251 bp), and two inverted repeats (IRa and IRb; 25,561 bp each). The overall GC content was 36.9% (LSC, 34.7%; SSC, 30.4%; IRs, 42.7%) and the plastome contained 130 genes, including 85 protein-coding, 8 rRNA, and 37 tRNA genes. A total of 19 genes were duplicated in the inverted repeat regions including eight tRNA, four rRNA, and six protein-coding genes. The complete *ycf1* gene was included in the IR at the SSC/IRa junction and the complete *infA* gene was located in LSC.

To confirm the phylogenetic position of *C. trigyna*, 21 representative species of Caryophyllales including 3 amaranth species and 7 of Chenopodiaceae were aligned using MAFFT v.7.388 (Katoh and Standley 2013) plug-in Geneious Prime v. 2019.1.3 (Biomatters, Auckland, New Zealand) and neighber-joining (NJ) analysis was conducted with *Phaulothamnus spinescens* (Achatocarpaceae) as an outgroup using Geneious Tree Builder of Geneious Prime v. 2019.1.3 (Biomatters, Auckland, New Zealand) and confidence for nodes determined using bootstrap analysis with 1000 replicates. The NJ consensus tree showed that the genus *Celosia* is closely related to the species in genus *Amaranthus*, and the four genera of family Amaranthaceae is closer with the subfamily Suaedoideae, Salicornioideae, and Suaedoideae than the subfamily Chenopodioideae in Chenopodiaceae (Figure 1).

**Figure 1.**
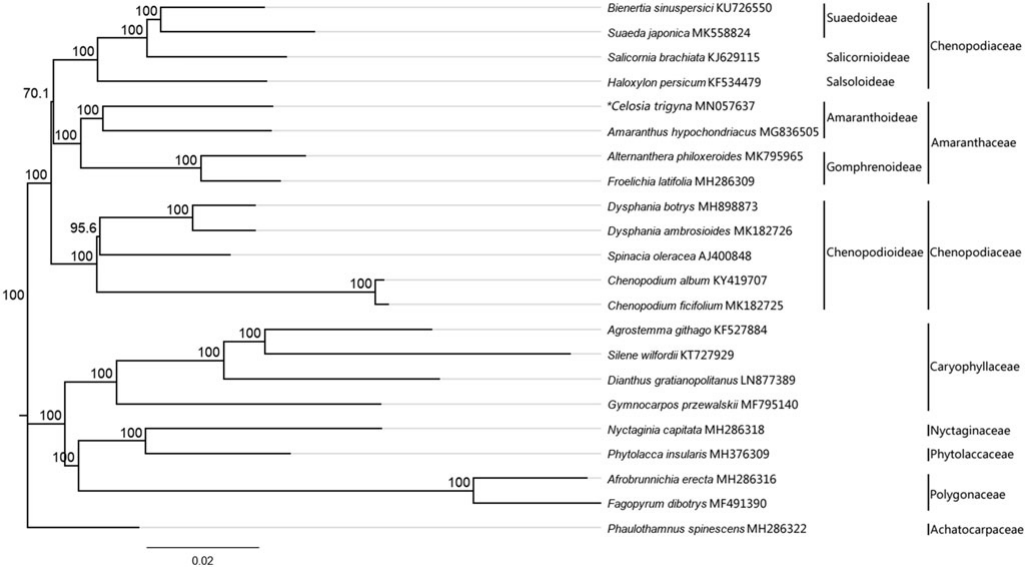
The neighber-joining (NJ) tree based on the 21 representative chloroplast genomes of order Caryophyllales. The bootstrap value based on 1000 replicates is shown on each node.
